# Hantavirus Infection in Humans and Rodents, Northwestern Argentina

**DOI:** 10.3201/eid0909.020768

**Published:** 2003-09

**Authors:** Noemi Pini, Silvana Levis, Gladys Calderón, Josefina Ramirez, Daniel Bravo, Elena Lozano, Carlos Ripoll, Stephen St. Jeor, Thomas G. Ksiazek, Ruben M. Barquez, Delia Enria

**Affiliations:** * Instituto Nacional de Enfermedades Virales Humanas “Dr. Julio I. Maiztegui,” Pergamino, Argentina; † Hospital San Miguel, Yuto, Jujuy, Argentina; ‡ Hospital Oscar Orias, Libertador General San Martín, Jujuy, Argentina; § Dirección de Epidemiología, Jujuy, Argentina; ¶ University of Nevada, Reno, Nevada, USA; # Centers for Disease Control and Prevention, Atlanta, Georgia, USA; ** Universidad Nacional de Tucumán, Tucumán, Argentina; †† Consejo Nacional de Investigaciones Científicas y Técnicas, Tucumán, Argentina

**Keywords:** Hantavirus, serology, humans, rodents, Hantavirus pulmonary syndrome, antibody

## Abstract

We initiated a study to elucidate the ecology and epidemiology of hantavirus infections in northern Argentina. The northwestern hantavirus pulmonary syndrome (HPS)–endemic area of Argentina comprises Salta and Jujuy Provinces. Between 1997 and 2000, 30 HPS cases were diagnosed in Jujuy Province (population 512,329). Most patients had a mild clinical course, and the death rate (13.3%) was low. We performed a serologic and epidemiologic survey in residents of the area, in conjunction with a serologic study in rodents. The prevalence of hantavirus antibodies in the general human population was 6.5%, one of the highest reported in the literature. No evidence of interhuman transmission was found, and the high prevalence of hantavirus antibody seemed to be associated with the high infestation of rodents detected in domestic and peridomestic habitats.

Hantaviruses (family *Bunyaviridae,* genus *Hantavirus*) are zoonotic viruses of rodents that produce two major clinical syndromes in humans: hemorrhagic fever with renal syndrome (HFRS) in Asia and Europe and hantavirus pulmonary syndrome (HPS) in the Americas. Since HPS was initially characterized in the United States in 1993 and the associated hantavirus (Sin Nombre virus, or SNV) was identified, an increasing number of human cases and SNV-related viruses have been identified in different countries of North and South America (*1*). Three HPS-endemic areas have been recognized in Argentina: northern (Salta and Jujuy Provinces), central (Buenos Aires, Santa Fe, and Entre Rios Provinces), and southern (Rio Negro, Neuquén, and Chubut Provinces). In the North, cases of acute respiratory distress syndrome of unknown etiology have been reported since 1984 at Orán, Salta Province. The illness, known in the area as ”Distress of Orán,” had an unexplained etiology until the early 1990s, when these cases were first associated with *Leptospira interrogans* infections and later with hantaviruses. Of 21 patients tested between 1991 and 1993, eight showed serologic evidence of recent leptospira infection by microscopic agglutination test, and 4 had a positive immunoglobulin (Ig) M enzyme-linked immunosorbent assay (ELISA) using Hantaan virus antigen (*2*,*3*). Ultimately, these patients were recognized as having HPS, and a new SNV-related hantavirus, now designated Oran virus, was recognized in the region (*4*). The first HPS cases in Jujuy Province were confirmed in 1997, and since then, their number has been progressively increasing. Isolated cases were detected in several different localities (San Pedro, La Mendieta, Caimancito, Libertador General San Martín, Fraile Pintado, and San Salvador, the provincial capital), but most originated in the town of Yuto and surroundings. A high percentage of confirmed cases had the usual nonspecific prodrome but were not followed by a distress syndrome. The case death rate (*4* [13.3%] of 30) was noticeably lower than that reported in other areas of the country and in the literature. Some strains of hantavirus were then hypothesized to produce subclinical disease. Only one hantavirus antibody-prevalence study had been performed among inhabitants of the Gran Chaco of Paraguay and Argentina (Salta Province), and hantavirus antibodies were found in 20% to 40% of participants (*5*).

Some differences in the clinical signs and symptoms of HPS have been recognized in other areas of the Americas compared with those described after infections with SNV; these differences have included possible person-to-person transmission, a different spectrum of clinical illnesses, an elevated incidence of infections in children, and higher antibody prevalence (*6*). For instance, patients from the area under study had unusually mild clinical symptoms and low death rates, supporting the idea that a less pathogenic hantavirus could be circulating in that area or that host or environmental factors might be responsible for the observed pattern. The objectives of this study were to determine the prevalence of hantavirus antibodies in the general population, identify risk factors, and investigate the rodent species implicated in hantavirus transmission in Yuto.

## Material and Methods

### Study Area

Yuto is located in the Ledesma Department, in the northeastern portion of Jujuy Province (23° 38′ S, 64° 28′ W). General topography is determined by the outlying spurs of the Andes range, and the area is covered by dense subtropical vegetation. The easternmost part of the study area is flat or slightly undulated, very fertile, with numerous rivers and streams and an average elevation of 349 m. Mean annual temperature is 20.7°C, ranging from 14.5°C in July (winter) to 25.8°C in January (summer). The rainy season starts in November as an annual monsoon, which lasts through the summer and into early fall; mean annual rainfall is 862 mm with a maximum monthly mean of 191 mm in January and a minimum monthly mean of 4 mm in July. Similar habitats and topography continue to the South (to Tucumán Province) and the North (to Oran, in Salta Province). The original biome of the area is a subtropical forest called “the yungas forests,” with numerous tree species of high economic value (*Anadenanthera colubrina, Calycophyllum multiflorum, Phyllostylon rhamnoides, Astronium urundeuva, Maclura tinctoria, Cordia trichotoma,* among others). This forest area is now considerably fragmented and modified by human agricultural activities. The main cultivated crop is sugar cane, which is grown from May to November. Other products include citrus fruits, avocados, pears, bananas, mangos, papayas, cherimoyas, and vegetables. Agriculture is the main source of employment, mostly involving manual labor. Housing for agricultural laborers is typically of very poor construction, in many cases consisting of shacks of salvaged wood and sheet metal. This type of domestic and peridomestic habitat offers prime conditions for rodent infestations, providing easy rodent access and poor sanitation, and is found even in the urban area of Yuto.

### Population Survey

**Figure 1:**
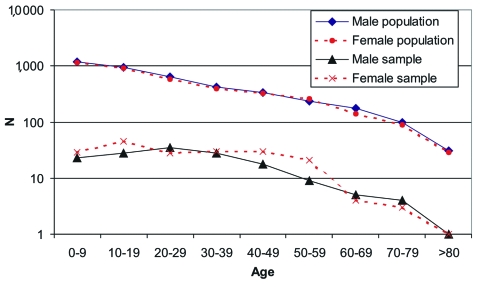
Distribution of general population and survey population by sex and age.

A cross-sectional study was performed on a sample of the general population of the area (population 7,900). The estimated sample size to document the overall prevalence in the total population was approximately 340 persons. Figure 1 shows the distribution of the general population and that of the survey participants by sex and age. Local physicians explained the objectives of the study to participants, and an informed consent agreement was signed by each person or by parents or legal guardians of minors. Each participant had a blood sample drawn and completed a questionnaire that covered personal data, ethnicity, household and workplace characteristics, occupation, domestic sightings of rodents, recreational activities, time of residence in the area, history of travel inside and outside the country, previous disease compatible with HPS, and contact with a confirmed HPS patient.

### Rodent Study

#### Trapping Site Selection

**Figure 2:**
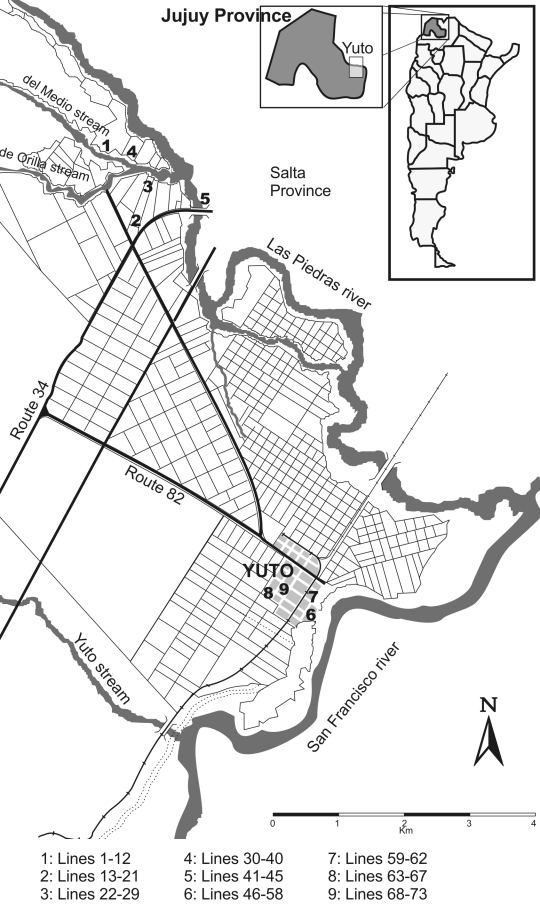
Localization of rodent trapping sites in Yuto and its surroundings.

Sherman live traps were placed at likely sites of exposure of previously documented HPS cases. Nine sites were selected: four sites in Yuto District (Guaraní [13 lines, 347 traps], Jardín [4 lines, 60 traps], 17 Has [4 lines, 124 traps], and 8 Has [7 lines,168 traps]); one in El Bananal, a small rural village 7 km outside Yuto (11 lines, 214 traps); three on or adjacent to farms (*fincas* [26 lines, 1,100 traps]); and one in a brushwood area (seminatural habitat [8 lines, 500 traps]). One farm was located in Urundel, Salta Province, in the immediate vicinity of Yuto, and the owner, workers, and inhabitants belonged to the Yuto community. Of the 73 capture lines, 19 were inside the household, 25 were peridomestic, 6 were in weeds near grapefruit culture, 5 in a brushwood, 5 at the side of a river or stream, 3 in vegetable gardens, 3 at roadsides, 2 in fruit orchards, 2 at the edge of a canal, and 3 adjacent to wire fences, railroads, or gullies. Outside lines consisted of 25 traps, each separated by 5 m. Lines located inside and outside the houses corresponded both to rural and urban areas. The number of traps inside the houses and in peridomestic urban lines depended on the area available at each site (8–20 traps). Figure 2 shows the location of trapping sites in Yuto and its surroundings.

### Trapping and Processing

Trapping was performed from May 30 to June 4, 2000. Small mammals were collected each morning and transferred to a field laboratory for processing. After being anesthetized with Isofluorane (Abbott Laboratories Ltd., Queenborough, England), animals were bled from the retroorbital sinus by using heparinized capillary tubes, and then killed by cervical dislocation while still anesthetized. Samples of serum, blood clot, brain, heart, kidney, liver, and lung were placed in cryovials and stored in liquid nitrogen for their subsequent analysis at the Instituto Nacional de Enfermedades Virales Humanas (INEVH). Carcasses were tentatively identified in the field and preserved in 10% formalin and sent to the Natural Sciences Museum “Miguel Lillo” in San Miguel de Tucumán for taxonomic confirmation. Small mammal trapping and processing were performed according to established safety guidelines (*7*).

### Serology

Human blood samples were centrifuged at Yuto Hospital. Serum was separated and placed in cryovials and stored in liquid nitrogen until further testing at INEVH. Rodent samples were centrifuged in the field laboratory and stored as described. Hantavirus antibodies were detected by an ELISA. Briefly, 96-well polyvinyl microplates were coated with SNV recombinant and control antigen overnight; then, serum samples and positive and negative controls were applied, followed by a peroxidase-conjugated antihuman IgG for human serum and a mix of peroxidase-conjugated anti–*Rattus norvegicus* and anti–*Peromyscus maniculatus* IgG for rodent serum. The substrate applied was 2,2′-azino-di (3-etilbentiazolin sulfonate) (ABTS, Kierkegaard & Perry Laboratories, Inc., Gaithersburg, MD). Serum dilutions were considered positive if the optical density was >0.2 after adjusting by subtraction of the corresponding negative-antigen optical density. Serum samples with titers >1:400 were considered positive.

## Results

### Serologic Survey in the General Population

Hantavirus IgG was found in 22 (6.5%) of 341 serum samples tested. For males, hantavirus antibody prevalence was 10%; females had a prevalence of 3.7%. Among the 341 participants, 56 were <10 years of age, 239 were 11–50 years of age, and 45 were >51 years of age (1 was without age data). Mean age among antibody-positive persons was 41 (range 18–87); 77% of these were in the 11- to 50-year age group. Hantavirus antibody prevalence according to sex and age are shown in Table 1.

**Table 1:** Serologic findings for hantavirus antibodies by age, sex

Age (y)	Females	**Males**
	**No. positive/tested (%)**	**No. positive/tested (%)**
**0–9**	**0/29 (0)**	**0/23 (0)**
**10–19**	**0/45 (0)**	**2/28 (7.1)**
**20–29**	**1/28 (3.6)**	**3/35 (8.6)**
**30–39**	**0/30 (0)**	**4/28 (14.3)**
**40–49**	**4/30 (13.3)**	**3/18 (16.7)**
**50–59**	**0/21 (0)**	**1/9 (11.1)**
**>60**	**2/8 (25)**	**2/10 (20)**
**Without data**		**0/1**
**Total**	**7/191 (3.7)**	**15/150 (10)**

Most (292/341, 85.6%) of the population in the survey were local born or native Argentinians. Twenty-five (7.3%) participants were foreigners, including 24 Bolivians and 1 Paraguayan. Twenty-two persons had aboriginal ancestors (6.5%), with 17 belonging to the Guarani community. No information was available for two persons. Only one of the aboriginal participants had IgG antibodies to hantaviruses (1 [4.5%] of 22). Table 2 shows hantavirus antibody prevalence among the study population by ethnicity or nationality.

**Table 2:** Ethnic demographics of the study population and hantavirus antibody prevalence^a^

**Ethnicity or nationality**	**No positive/tested (%)**
**Native^b^ without specification**	**13/229 (5.7)**
**Native with Bolivian lineage**	**6/56 (10.7)**
**Native with Paraguayan lineage**	**1/7 (14.2)**
**Native total**	**20/292 (6.8)**
**Indigenous without specification**	**0/3 (0)**
Indigenous Guaraní	**0/17 (0)**
Indigenous Chaguanco	**0/1 (0)**
**Indigenous Charagua (Bolivia)**	**1/1 (100)**
**Indigenous total**	**1/22 (4.5)**
**Bolivians**	**1/24 (4.2)**
**Paraguayan**	**0/1 (0)**
**Foreigners total**	**1/25 (4)**
**Without data**	**0/2 (0)**

**^a^By immunoglobulin G enzyme-linked immunosorbent assay.** **^b^Native, born in Argentina.**

Dwellings were characterized according to their location as urban (>500 m from an open field), suburban (50–500 m from an open field), and rural (<50 m from an open field). Table 3 shows hantavirus antibody-prevalence findings in relation to house location and occupation of participants. Forty persons with urban occupations included 10 administrative employees, 13 health workers, 5 housewives, 4 students, and other miscellaneous occupations (technician, gardener, bricklayer, retired). Among suburban study participants, 23 were housewives, 28 were students, and the rest were employed in a variety of occupations (employee, health agent, maid, bricklayer). Among rural participants, 75 were agricultural workers, 64 housewives, 29 students, 10 sawmill workers, and the rest had miscellaneous occupations (employee, trader, bricklayer, retired). All three hantavirus-antibody–positive participants living in urban dwellings worked in rural areas (a bricklayer, a sawmill worker, and an agricultural worker), as did two antibody-positive participants living in suburban houses (agricultural workers). Thirteen (17.1%) of 76 participants with antibodies were agricultural workers (laborers, farmers, *fincas* owners). Antibody prevalences for other occupations included housewives (4 [6.3%] of 64), and sawmill workers (1 [10%] of 10). We found no hantavirus antibodies among 61 students or 20 healthcare workers, including physicians, nurses, health agents, and a dentist.

**Table 3:** Hantavirus antibody prevalence by location of residence site and location of occupational activity^a^

	**Urban** **No positive/tested (%)**	**Suburban** **No positive/tested (%)**	**Rural** **No positive/tested (%)**
**Dwellings**	**3/31 (9.7)**	**4/121 (3.3)**	**15/189 (8)**
**Occupational activity**	**0/40 (0)**	**1/57 (1.8)**	**20/201 (10)**

**^a^As determined by immunoglobulin G enzyme-linked immunosorbent assay.**

If occupations are considered as rural (positive IgG, 20 [10%] of 201) or not rural (urban and suburban, positive IgG 1 [1.03%] of 97), hantavirus antibody prevalence is significantly higher in the former (chi square=7.95, p=0.004); among those with rural occupations, those whose employment included agricultural activities had a higher prevalence of hantavirus antibodies (positive IgG 13 [17.1%] of 76; 7 [5.6%] of 125; chi square=6.98, p=0.008).

Table 4 shows clinical and epidemiologic data for the study population. Most (86%) hantavirus antibody–positive participants did not recall previous HPS clinical manifestations. The presence of rodents was reported by 77% of hantavirus antibody–positive and 79% of hantavirus antibody–negative persons, both in peridomestic and workplace settings.

**Table 4:** Hantavirus antibody prevalence by clinical and epidemiologic characteristics

	**Yes**	**No**	**Chi square,^a^ p value**	**Without data**	
	**Positive/tested (%)**	**Positive/tested (%)**		**Positive/tested (%)**	
**Previous clinical HPS^b^ symptoms**	**3/29 (10.3)**	**19/307 (6.2)**	**0.75, 0.38**	**0/5 (0)**	
**Contact with a confirmed HPS case-patient**	**6^c^/98 (6.1)**	**16/242 (6.6)**	**0.03, 0.86**	**0/1 (0)**	
**Recreational activities (fishing, hunting)**	**6/77 (7.8)**	**16/260 (6.2)**	**0.26, 0.60**	**0/4 (0)**	
**Sighting of rodents**	**17/270 (6.3)**	**5/71 (7.0)**	**0.05, 0.81**		
**Trips outside the area**	**15/233 (6.4)**	**7/105 (6.7)**	**0.01, 0.93**	**0/3 (0)**	

**^a^Chi-square test for comparison of two proportions in two independent samples, p>0.05 no significance. EpiInfo version 2000 (Centers for Disease Control, Atlanta, GA).** **^b^HPS, hantavirus pulmonary syndrome.** **^c^All positive contacts were relatives of a confirmed HPS patient.**

Among persons who had previous contact with known HPS patients, 6 (6.1%) of 98 cases had hantavirus antibodies. Similar antibody prevalence was found in persons who did not have prior contact with a known HPS patient (16 [6.6%] of 242; chi square p>0.05). One hundred five persons (30.8%) reported no trips outside the area, and 58% of the remainder had traveled only to other areas inside the province or to nearby Salta Province; 41.6% reported trips to Bolivia in addition to local trips. Only a small percentage (0.4%) had visited relatively distant areas of the country. Hantavirus antibodies were found in 15 (6.4%) of 233 persons who traveled outside the immediate region, and 7 (6.6%) of 105 who had not traveled outside of the immediate region.

### Rodent Study

A total of 361small mammals were captured in 2,427.5 trap-nights (overall trap success 14.8%). Captures represented two rodent families, three subfamilies, and 13 species (Table 5). *Calomys* and *Akodon* were the most frequently trapped genera (38% and 40.2%, respectively) and the sole taxa that were found with hantavirus antibodies. Hantavirus IgG was found in 4 (2.8%) of 140 *Akodon simulator* and 7 (5.1%) of 137 *Calomys callosus.* The genus *Oligoryzomys* has several species previously identified as hantavirus reservoirs in the three HPS-endemic areas of the country. In this study, this genus accounted for 12 (3.3%) of the 361 captures; however, none was positive for hantavirus antibody. Table 6 shows the species distribution according to the different habitats sampled. The specimens of *C. callosus* were trapped inside dwellings located at different *fincas*.

**Table 5:** Relative abundance and hantavirus antibody prevalence of rodent species in Yuto (Jujuy Province)

**Order**	Family	Subfamily	Genus/species	**Captures (%)**	**No. positive/tested(%)**
Rodentia	*Muridae*	Sigmodontinae	*Akodon simulator*	**140 (39.0)**	**4/140 (2.8)**
			*A. caenosus*	**3 (0.8)**	**0/3**
			*Akodon sp*	**2 (0.6)**	**0/2**
			***Calomys callosus***	**137 (38.0)**	**7/137 (5.1)**
			***Holochilus chacarius***	**11 (3.1)**	**0/11**
			***Oligoryzomys chacoensis***	**4 (1.1)**	**0/4**
			***O. longicaudatus***	**6 (1.7)**	**0/6**
			***Oligoryzomys sp***	**2 (0.6)**	**0/2**
			***Oxymycterus paramensis***	**3 (0.8)**	**0/3**
		**Murinae**	***Mus musculus***	**10 (2.8)**	**0/10**
			***Rattus rattus***	**6 (1.7)**	**0/6**
**Didelphimorphia**	***Didelphidae***	**Marmosinae**	***Thylamys venustus***	**2 (0.6)**	**0/2**
			***T. elegans***	**1 (0.3)**	**0/1**
**No identification**				**34 (9.4)**	**0/34**
**Total**				**361 (100)**	**11/361(3.0)**

**Table 6:** Distribution of rodent species by capture habitat

**Habitat**								
**Species**	**Districts**		**Farms**			**Brushwood^d^**	**Total**	
	**Domestic^a^**	**Peridomestic^b^**	**Domestic^a^**	**Peridomestic^b^**	**Cultivation^c^**			
***Akodon simulator***		**5**		**7**	**51**	**77**	**140**	
***Akodon sp***						**2**	**2**	
***Akodon caenosus***						**3**	**3**	
***Calomys callosus***		**8**	**5**	**25**	**44**	**55**	**137**	
***Holochilus chacarius***					**7**	**4**	**11**	
***Mus musculus***	**7**	**3**					**10**	
***Oligoryzomys sp***					**2**		**2**	
***Oligoryzomys chacoensis***						**4**	**4**	
***O. longicaudatus***						**6**	**6**	
***Oxymycterus paramensis***		**1**				**2**	**3**	
***Rattus rattus***		**1**	**4**	**1**			**6**	
***Thylamys elegans***					**1**		**1**	
***T. venustus***		**1**			**1**		**2**	
**No identification**		**1**			**17**	**16**	**34**	
**Total**	**7**	**20**	**9**	**33**	**123**	**169**	**361**	

**^a^Inside households.** **^b^Immediate vicinity of houses, including yards, outbuildings, vegetable gardens, weeds, fence lines, and railroad.** **^c^Includes grapefruit and banana plantations, vegetables cultivation, weeds, and roadside in the same sites.** **^d^Seminatural habitat that includes a streamside.**

More than half (6 [54.5%] of 11) of the positive rodents were captured in weeds, roadsides, or peridomestic sites at *fincas* (fruit trees and vegetable plantations); four were trapped in brushwood, and the last one at the riverside very near an HPS patient’s dwelling at El Bananal. Two lines contributed with two positive rodents on each. These were located in grapefruit plantations (weeds and roadside).

## Discussion

The differences observed in the South American hantavirus infections relative to the classical SNV-related syndrome in North America have been suggested to reflect approaches to surveillance and differences in the pathogenicity of the viruses, host-reservoirs, and ecologic factors. The particular pattern of mild clinical illnesses and low case-fatality rate found in Yuto determined our selection of the area for detailed studies. In this first investigation, we attempted to determine the prevalence of past infection in the general population by testing for hantavirus IgG antibodies, to identify risk factors, and to identify the rodent species implicated in the transmission of hantaviruses

The hantavirus antibody prevalence found in the human population survey is one of the highest reported in Argentina, with a mean of 6.5% (females 3.7%, males 10%). Previous studies in the other HPS-endemic areas (central and southwestern) of Argentina found antibody prevalence varying from 0.1% to 1.5% (*4*,*8*). Males in their 30s and 40s showed antibody prevalences of >14% and 16%, respectively. Most antibody carriers (82%) did not report clinical manifestations consistent with HPS. Thus, the low case-death rate clearly reflects milder clinical illnesses (reported case-fatality rates are 40% to 50% in both Americas) (*9*,*10*).

In previous studies of asymptomatic contacts of HPS case-patients from this area, we found a high prevalence of IgG (4 [9.5%] of 42). This finding could be the result of infection from a common source or interhuman transmission (*6*), as described in the 1996 outbreak in El Bolson-Bariloche, southern Argentina (*11*–*13*). In this survey, we did not find differences in the hantavirus antibody prevalence in persons with and without known HPS case contact (6.1% and 6.6%, respectively). No antibodies were found among the healthcare workers studied, and the distribution of clinical cases and antibody carriers by sex showed a predominance of males (in patients from 1997 to 2000, the percentage of males was 76.7%, 23/30). These findings, collectively, do not favor the hypothesis that interhuman spread is playing a large role in the transmission of hantaviruses in this area. These findings also reinforce the view that environmental, occupational, and residential factors create an increased risk for rodent exposure in occupational, domestic, and peridomestic settings. This conclusion is supported by the noticeable observation of rodents and their signs in households and workplaces reported by the study population and patients. The risk factor that showed a significant difference between antibody-positive and -negative persons was a rural occupation, especially one associated with agricultural activities. This finding was also reflected in the high male antibody prevalence observed in the survey and predominance of males among patients.

A high antibody prevalence has been previously found in indigenous communities of the Gran Chaco of Paraguay (40.4%) and Argentina (17.1%) (in Salta Province). In those studies, the aboriginals evaluated belong to closed communities that still fish, hunt, and gather for their sustenance. Their main ethnic groups are Chorote, Chulupi, and Wichi of the Mataco-Mataguayan linguistic family (*5*). In the area in our study, aboriginal and foreign people are in the minority (14% in the sample). Indigenous people (22 in this sample) belong to different groups; 77% are Guarinis from Paraguay. Only one person from the Charagua community, which originated in Bolivia, participated in the study. This person had hantavirus antibodies. Among the 25 foreign participants, the only antibody-positive person belonged to the Bolivian majority (*14*). All nonnative inhabitants, including those with aboriginal ancestors and foreigners, are integrated members of the general population, sharing jobs and household conditions with local people, and therefore sharing similar risk factors.

More than two thirds of the studied group had traveled inside or outside the province, and/or to Bolivia. Such trips are frequent among migrant farm laborers, who follow harvest seasons. No differences were found in the antibody prevalence between persons who traveled and those who did not, probably because the reported trips were primarily within the same ecologic area.

The genetic diversity of sigmodontine rodents in South America is well known (*15*). Characterization of rodent species and their association with indigenous hantaviruses are currently under study. Putative rodent reservoirs of pathogenic hantaviruses identified in Argentina thus far belong to the *Oligoryzomys* genus (*O. longicaudatus* for Andes and Oran genotypes, *O. chacoensis* for Bermejo genotype, and *O. flavescens* for Lechiguanas genotype). Three previous rodent expeditions were performed in the northwestern Argentine hantavirus-endemic area in relation to HPS studies: two in Salta Province (July 1995 and October 1996) and one in Jujuy Province (May 1998), involving the villages of La Mendieta and Libertador General San Martin. Hantavirus antibody–positive species from Salta included *O. chacoensis* (1 [3.7%] of 27), *A. simulator* (1 [3.8%] of 26), and *O. longicaudatus* (2 [7.7%] of 26) and in Jujuy *O. chacoensis*, (1 [8.3%] of 12) (*16*).

In the present study, hantavirus antibody prevalence among rodents was similar to that previously reported in the country (2.7% to 12.5%, varying by area and species) (*16*), but the species found with hantavirus antibodies were different. The genera of hantavirus antibody–positive rodents corresponded to those with higher relative abundance, *Akodon* and *Calomys*. *Akodon,* associated with the Pergamino virus in central Argentina, has thus far not been reported to be pathogenic for humans (*17*). Among the species of *Calomys*, *C. laucha* has been identified as the reservoir of Laguna Negra virus in Paraguay, but no previous evidence suggests it circulated in Argentina (*14*). Sigmodontine rodents were collected in every rural habitat in which we used traps, including inside dwellings, peridomestic sites, weeds close to grapefruit and banana plantations, vegetable fields, and mainly natural habitats such as woodbrush and the sides of rivers and streams. Most positive rodents were captured in weeds or roadsides inside or close to cultivated citrus or vegetables. Other rodents were captured in peridomestic sites associated with HPS cases and in woodbrush near one of the *fincas*. Focal concentration of positive rodents appeared to occur, with multiple positive rodents often trapped in the same trap line.

Characteristics of household or working habitats included a great deal of available potential food and cover for rodents attributable to substandard housing and sanitation. Sigmodontine rodents were also trapped in peridomestic sites of the urban area (8 Has and 17 Has quarters), where the features of the environment and buildings were similar to suburban or rural areas. *C. callosus* was the only wild rodent species captured inside houses. This observation is in accord with previous descriptions from Bolivia in relation to the Bolivian hemorrhagic fever outbreaks; *C. callosus* is the reservoir of Machupo virus, the arenavirus linked to Bolivian hemorrhagic fever, in the BHF-endemic area of El Beni (*18*). Control of the large Bolivian hemorrhagic fever outbreaks of the 1960s was achieved through measures directed to prevent infestation of *C. callosus* in towns and villages. These same measures should also be useful in this area to prevent hantavirus transmission, at least from rodents of the genus *Calomys* that are adapted to domestic and peridomestic settings.

Our results favor the hypothesis that less virulent hantaviruses are responsible for the mild and subclinical illnesses circulating in this region. Ongoing investigations that include the genetic characterization of the viruses associated with the different clinical forms will help to clarify this point.

## References

[1] Peters CJ, Simpson GL, Kevy H. Spectrum of hantavirus infection: hemorrhagic fever with renal syndrome and hantavirus pulmonary syndrome. Annu Rev Med. 1999;50:531–45. 10.1146/annurev.med.50.1.531 10073292

[2] Cortes J, Cacace ML, Seijo A, Parisi MN, Ayala LT. Distress respiratorio del adulto en Orán, Salta. I Congreso Interamericano de Infectología. Córdoba, Argentina, 1994. Libro de resúmenes. p. 145.

[3] Riera LM, Parisi MN, Seijo A, Pini NC, Sabattini M, Enria DA. Infección por Leptospira y virus Hantaan en pacientes con fiebre hemorrágica en el área endémica de FHA. I Congreso Interamericano de Infectología. Córdoba, Argentina, 1994. Libro de resúmenes. p. 148.

[4] Levis SC, Briggiler AM, Cacace M, Peters CJ, Ksiazek TG, Cortes J, Emergence of hantavirus pulmonary syndrome in Argentina. Am J Trop Med Hyg. 1995;53(Suppl):233.7573702

[5] Ferrer JF, Jonsson CB, Esteban E, Galligan D, Basombrio MA, Peralta-Ramos M, High prevalence of hantavirus infection in Indian comunities of the Paraguayan and Argentinean Gran Chaco. Am J Trop Med Hyg. 1998;59:438–44. 9749641 10.4269/ajtmh.1998.59.438

[6] Enria DA, Briggiler AM, Pini N, Levis S. Clinical manifestations of New World hantaviruses. In: Schmaljohn CS, Nichol ST, editors. Hantaviruses. New York: Springer-Verlag; 2001. p. 117–34. 10.1007/978-3-642-56753-7_7 11217400

[7] Mills JN, Childs JE, Ksiazek TG, Peters CJ. Methods for trapping and sampling small mammals for virologic testing. Atlanta: U.S. Department of Health and Human Services, Centers for Disease Control and Prevention; 1995.

[8] Wells RM, Sosa Estani S, Yadon Z, Enria D, Padula P, Pini N, Seroprevalence of antibodies to Hantavirus in health care workers and other residents of southern Argentina. Clin Infect Dis. 1998;27:895–6. 10.1086/517161 9798052

[9] Khan AS, Khabbaz RS, Armstrong LR, Holman RC, Bauer SP, Graber J, Hantavirus pulmonary syndrome: the first 100 US cases. J Infect Dis. 1996;173:1297–303. 8648200 10.1093/infdis/173.6.1297

[10] Lazaro ME, Resa AJ, Barclay CM, Calanni L, Samengo L, Martinez L, Sindrome pulmonar por hantavirus en el sur andino argentino. Medicina (B Aires). 2000;60:289–301. 11050803

[11] Enria DA, Padula P, Segura EL, Pini N, Edelstein A, Posse CR, Hantavirus pulmonary syndrome in Argentina. Possibility or person-to-person transmission. Medicina (B Aires). 1996;56:709–11. 9284576

[12] Wells RM, Sosa Estani S, Yadon Z, Enria D, Padula P, Pini N, An unusual hantavirus outbreak in southern Argentina: person-to-person transmission? Emerg Infect Dis. 1997;3:171–4. 10.3201/eid0302.970210 9204298 PMC2627608

[13] Padula P, Edelstein A, Miguel SDL, Lopez NM, Rossi CM, Rabinovich RD. Hantavirus pulmonary syndrome (HPS) outbreak in Argentina: molecular evidence for person-to-person transmission of Andes virus. Virology. 1998;241:323–30. 10.1006/viro.1997.8976 9499807

[14] Johnson AM, Bowen MD, Ksiazek TG, Williams RJ, Bryan RT, Mills JN, Laguna Negra virus associated with HPS in western Paraguay and Bolivia. Virology. 1997;238:115–27. 10.1006/viro.1997.8840 9375015

[15] Plyusnin A, Morzunov SP. Virus evolution and genetic diversity of Hantaviruses and their rodent hosts. In: Schmaljohn CS, Nichol ST, editors. Hantaviruses. New York: Springer-Verlag; 2001. p. 47–75. 10.1007/978-3-642-56753-7_4 11217406

[16] Calderon G, Pini N, Bolpe J, Levis S, Mills JN, Segura E, Hantavirus reservoir hosts associated with peridomestic habitats in Argentina. Emerg Infect Dis. 1999;5:792–7. 10.3201/eid0506.990608 10603213 PMC2640793

[17] Levis S, Morzunov S, Rowe J, Enria D, Pini N, Calderon G, Genetic diversity and epidemiology of hantaviruses in Argentina. J Infect Dis. 1998;177:529–38. 10.1086/514221 9498428

[18] Johnson KM. Emerging viruses in context: an overview of viral hemorrhagic fevers. In: Morse S, editor. Emerging viruses. New York: Oxford University Press; 1993. p. 52.

